# Deconstructing Psychedelic Phenomenology: A Thematic Analysis of Discrete Phases of the Psychedelic Experience

**DOI:** 10.1002/brb3.70687

**Published:** 2025-07-21

**Authors:** H. T. McGovern, L. Bajo, G. Hassed, R. Hoefnagels, M. Levey, N. Rose, A. De Foe, B. T. Hutchinson

**Affiliations:** ^1^ IMPACT – Institute for Mental and Physical Health and Clinical Translation, School of Medicine Deakin University Geelong Victoria Australia; ^2^ School of Psychology The University of Queensland St Lucia Queensland Australia; ^3^ School of Educational Psychology and Counselling Monash University Clayton Victoria Australia; ^4^ Faculty of Behavioural and Movement Sciences, Cognitive Psychology Vrije Universiteit Amsterdam Amsterdam The Netherlands

**Keywords:** eudaimonia, experience, health psychology, psychedelics, qualitative methods

## Abstract

The phenomenology of psychedelic experiences has been a long‐standing point of interest to researchers. However, internal experience has been relatively relegated, with much work done on the clinical outcomes of psychedelic therapies. Our reflexive thematic analysis revealed that structurally, people on fora write about their experiences sequentially, considering factors prior to (preparatory), during (acute phase), and after their account of ingesting psychedelics. Themes constructed prior to experience were (1) subjective knowledge and perception of psychedelics, (2) intention and efforts to mentally prepare, and (3) experiential aids. Generated themes during the experience were (1) sensory and cognitive distortions, (2) mindset and affective quality, and (3) environmental stability and support. Experiential impact on behavior and outlook was constructed as the unitary theme following the experience. Future work should look more closely at the role of set and setting within the context of the stages leading up to, during, and after psychedelic administration.

## Graphical abstract

A thematic analysis of psychedelic experiences was partitioned into three temporal phases: before, during, and after the experience. Themes before the experience were preparatory intentions and perceptions; during the experience, acute sensory and affective distortions; and after the experience, changes in behavior and outlook.

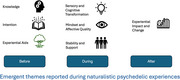

## 1 Introduction

Psychedelics have generated interest based on emergent evidence of clinical efficacy (Hadar et al. 2023). One area requiring elucidation is how people construct meaning from psychedelic experiences and interpret outcomes that come from the experience. This is important, as increasing media attention and public awareness are contributing to public interest and increased underground consumption (Kruger et al. 2023).

Despite their potential, responses to psychedelics can vary (Haijen et al. 2018). While many document positive effects (Vollenweider and Preller 2020), others report aversive experiences (Schlag et al. 2022), which are associated with worse long‐term mental health outcomes (Bienemann et al. 2020). This variability complicates clinical implementation (Aday et al. 2021; McCartney et al. 2023), necessitating attention to factors such as set (i.e., mindset; Noorani 2021) and setting (i.e., environment and social context; Roberts et al. 2020), which shape experiential trajectories (Leary et al. 1963; Hartogsohn 2017).

Recent work emphasizes the importance of nonpharmacological factors for experiential quality (Gukyasan and Nayak 2022). Haijen et al. (2018) found that clear intentions and lower baseline anxiety and environmental familiarity reduced challenging experiences. However, little work has focused on qualitative aspects of set and setting. McCartney et al. (2023) utilized online forums and identified themes including prior knowledge of psychedelics (i.e., informed versus uninformed), mindset (i.e., surrendered, or resistant mindset), and motivation (i.e., escapism or self‐exploration motives) (also see Pronovost‐Morgan et al. 2023) that influenced psychedelic experiences.

We build on prior work by drawing on a larger and more diverse dataset. The introduction of temporal partitioning enabled us to construct a more nuanced and phase‐specific thematic structure, capturing how participants made sense of each stage of the experience. This, in turn, offers a more process‐oriented account of meaning‐making within naturalistic psychedelic use.

## 2 Methodology

### 2.1 Procedure

Twenty first‐person written reports of psychedelic experiences from the online databases of Reddit^1^, Shroomery, and Psychonaut Wiki were collected (three from Shroomery, 11 from Reddit, and six from Psychonaut Wiki). Data on these platforms functioned akin to archival data; therefore, no proactive enlistment of participants took place. To ensure anonymity, any demographic or otherwise potentially identifying information (such as through posts or individual accounts) was not included. Ethics exemption was granted by the University of Queensland ethics and integrity committee (ethics ID: HE001679).

## 3 Analysis and Results

Consistent with our reflexive analysis approach, we reflected through the analysis process on how positionality influenced the analytic process. We all carry academic backgrounds in psychology, and we are all familiar with the relevant research literature on psychedelics. Such backgrounds influenced our interpretation and sensitivity to the data. Through the analytic process, we conducted regular discussions to critically examine our assumptions and how our backgrounds informed our development of themes (see Supporting Information for full positionality statement and methods). Set and setting considerations were prominent in the before/during phases. “Before” phase themes included knowledge, intentionality, and experiential aids. “During” phase themes encompassed internal factors (sensory and mindset) and external elements (environmental stability and social support). “After” phase reports focused primarily on behavioral and attitudinal impacts with fewer explicit internal/external distinctions. Figure 2 illustrates themes across all phases.

### 3.1 Before

#### 3.1.1 Theme 1: Subjective Knowledge (or Lack of) and Perception of Psychedelics

A generated theme concerned the participant's subjective psychedelic knowledge, shaping perceptions and willingness to consume them. This knowledge reflected what they believed they had learned about psychedelics prior to their experiences.

One report notes, “I had researched mushrooms and was extremely interested in them for years” (Report 19), and “I had been researching psilocybin after learning about the potential therapeutic benefits” (Report 13); another notes more experiential knowledge, “I figured since I've done mushrooms about ten times prior, it would not be a problem” (Report 2). Others seemed to have more spontaneous experiences, not informed by experiential knowledge “… I remember one day she asked me if I wanna do acid with her for the first time, we had never done it before” (Report 4).

Participants entered the experience with varying levels of academic and experiential knowledge. Some experiences were spontaneous; therefore, prior knowledge was not an influential factor. More subjective psychedelic knowledge seemed to influence decisions to take psychedelics, consistent with past research (Kettner, Mason, et al. 2019), attitudes (Nadeem et al. 2024), and experiences (Haijen et al. 2018). This theme also pointed to the fact that prior knowledge seemed to influence expectations around the experience, “I have previous experience with LSD (100 µg, 150 µg), and Psilocybin…, so I was confident that I would enjoy it” (Report 1). More knowledge, particularly based on past consumption, was often associated with a tendency to perceive challenging aspects of the experience as either meaningful or as potential to generate insight, “I begin feeling intense feelings of dread and that I was no longer in control of the situation or that I was enjoying it…… realized that this may be a moment that could lead to something good” (Report 1).

#### 3.1.2 Theme 2: Intention and Efforts to Prepare

This theme reflects how intentions to take psychedelics were informed and how people prepared. Often linked, intention and preparation could also diverge. Some carried into the experience an explicit therapeutic intention, “I needed to have a shroom trip as I find it very useful to work through things” (Report 12), and “I set my intentions for the trip” (Report 12), while others carried recreational or escapist intentions, “A few good friends and I had a plan to take 3 g of mushrooms and hang out” (Report 3), and “I had nothing better to do” (Report 9). Knowledge of psychedelics seemed to influence the subjective sense of preparation, “My set and setting seemed good, I was in a good mindset, I knew what to expect” (Report 19).

We interpreted a push and pull relationship between preparing and intention setting, “Before I go in depth on the trip, I want to say the precautions set for the trip were close to none. We didn't have a trip sitter and it was somewhat of a spontaneous idea that had no mental preparation” (Report 5). Report 5 notes more knowledge and equates the spontaneity of the experience with a lack of preparation. Reporter 3 seemingly accumulated therapeutic intention through academic psychedelic knowledge, “I have been interested in psychedelics for years. After some talks with friends, I finally decided to dip my toes in” (Report 3), enabling intention to form despite the spontaneity.

Intention and preparation efforts thus seemed interconnected, with reporters noting meaningful experiences required mental efforts (e.g., establishing clear intentions and purpose). Academic or experiential knowledge may support preparation, even for spontaneous consumption. These findings align with research showing links between preparation, intention, and experiential quality (Hartogsohn 2017; Timmermann et al. 2022).

#### 3.1.3 Theme 3: Experiential Aids

Another generated theme was the use of experiential aids, which could amplify intensity and shift experiences from negative to positive affect, consistent with past work (McCartney et al. 2023). These aids fall under the remit of set and setting (factors in the environment shaping the experience [Hartogsohn 2017]). The most common aids here were music, nature, and having a sitter.

Music seemed important for preparation, “I put on some noise‐cancelling bluetooth headphones and started listening to Skeggs. I ended up leaving the fairly short This is Skeggs playlist on Spotify on repeat for most of the evening” (Report 10), “…we decided to shut our eyes and listen to music” (Report 6), “I started a playlist of music…” (Report 1), and trying to ensure a more immersive subjective experience, “…I'm tripping hard and trying to sink in to the experience” (Report 19). This was also the case with reporters choosing to be in nature, “I lay down in the grass, admiring the trees and cloudy sky” (Report 13), and “Despite the short notice, and little sleep, I agreed, figuring it would be an ideal time for some nature and shrooms” (Report 2), and having a sitter, “I put the other half away and lay down on the bed with my partner. We watch YouTube while I wait for it to hit” (Report 3).

Participants seemed to view experiential aids as tools amplifying intensity and deepening immersion. Nature in music particularly enhanced the experiences and increased apperception of the aids themselves. This appeared to be a reciprocal process, where the experiential aids facilitated deeper immersion, which in turn enabled more engaged and meaningful encounters with the experience. Likewise, sitters seemed to provide a means of immersion via providing support, and using sitters could solicit greater connection and emotional vulnerability with the sitter, “I texted my wife and asked her to come in and help to ground me. She held me for a couple of hours as I just poured my heart out to her” (Report 11).

While each of the environmental factors above served as environmental aids (i.e., music, being alone at home), being in natural settings seemed to confer the highest degree of support, as its characteristics most consistently provided a metaphor through which people described positive psychological insights. The experiential aids seemed to provide an impetus toward a greater sense of insight, “The power of nature just IS, and nothing I do can possibly change how it acts. I am helpless…It makes no sense to try to change the weather, you just have to GO WITH IT. Go with the flow. Adapt and accept” (Report 11). In other cases, nature provided an impetus toward meaningful action or life changes during the experience, “And then, I felt an immense craving for food with WATER in it. I just wanted tomato, fruits, salad” (Report 13). Characteristics of nature thus seemed to become the object of desired change (e.g., become more like water), while in other cases, it was used as something people wished to become more immersed in. In either case, nature and its characteristics seemed to provide a language or metaphor through which people could describe their psychological change. Our findings align with research showing music (Kaelen et al. 2018), nature (Gandy et al. 2020; Kettner et al. 2018), and sitters (Thal et al. 2022) can amplify and shift experiences. These aids appear essential to subjective experiences, potentially because they work synergistically with psychedelics to enhance sensory immersion and subjective intensity.

### 3.2 During

#### 3.2.1 Theme 1: Sensory and Cognitive Transformation

Sensory and cognitive transformation was a core theme, often producing insights that transformed subjective understanding. These insights could emerge through beings:

I immediately saw giant pyramids morphing into each other with a flaming elk skull face. It turned around and showed me what looked like an infinite energy swirling and moving atoms with it. I stared at it for what felt like forever and I slowly drifted towards it I felt myself being absorbed into it… Last thing I saw was it just looking at me in what felt like a taunt. Literally felt like it was saying “this is how it is what are u going to do about it”. Seak nothing or stay as you are and be able to enjoy what u have (Report 6).

These insights could also emerge through internal sense of knowing, “I became aware of the absurdity of many of the societal patterns we impose upon each other as humans” (Report 17), and “this was an experience of a new sense of joy, deeper than anything the child had ever felt before” (Report 15), and were often revelatory in nature, “I have an epiphany that much of my whole life has been an ongoing pursuit of the next high—either food, video games, friends, sex, whatever. It is all a pursuit of meaningless, chemical fixations of serotonin or dopamine. I remind myself that I am tripping right now and I shouldn't worry too much about any thoughts I am having” (Report 3).

Reports often converged on sudden revelations or insights as central features, arising in different ways. Sensory disturbances were often conduits for these insights and were attributed high epistemic value. Realizations about everyday meaninglessness provided relief and profundity and shifted experiences toward positive affectivity. This is consistent with work showing that psychedelics induce insight and shifts in self‐concept and worldview (Gandy et al. 2022; Tulver et al. 2023), and with notes that realizing futility often precedes psychological breakthroughs (Letheby and Gerrans 2017).

#### 3.2.2 Theme 2: Mindset and Affective Quality

Another theme concerned participants’ mindset and affective quality. A dynamic tension emerged between surrender (associated with positive experiences) and resistance (linked to negative experiences). Surrender appeared crucial for positive states, perhaps because letting go allowed experiences to unfold naturally rather than through the analytical lens of ordinary consciousness.

Some reports outlined being able to “surrender” to the experience, “I am ready and accepting of whatever the experience will be” (Report 3). One report demonstrates a sense of surrender not only to the experience but also to a more general relinquishing of the boundary between self, conscious experience, and the universe—they just *were* the experience: “I felt myself being absorbed into it. Like my consciousness had dispersed and become the universe itself, but I no longer existed. I no longer felt or thought anything. I was nothing and everything. I was every atom in existence but I wasn't me. It was peaceful (Report 6).”

Other reports reported more resistance to the experience, “I'm only aware of my quickly dissolving ‘self’/ego, and the torrent of my negative subconscious that I fear will overwhelm me if I surrender to it… I start thinking about how this isn't what I wanted, not what I hoped to get” (Report 19). These findings are consistent with the more general concept of ego‐dissolution, an important aspect of the more eudaimonic types of subjective psychedelic experience (Letheby and Gerrans 2017), and with the potential held for a departure in subjective experience.

#### 3.2.3 Theme 3: Environmental Stability and Support

Stability and support was a central theme. This refers to whether someone was in a stable (i.e., the same) environment through the acute phase, and whether they had support. Having relatively more support and environmental stability seemed to encompass an ability to fully relax during the experience, “I felt like I had peaked and when I felt it coming down a little he was mellowing out a lot and we decided to shut our eyes and listen to music” (Report 6), although support could also be conferred socially, even when environments were unstable: “When that happened we headed over to a pub because they had a folk night event” (Report 20). Stable environmental settings may allow a sense of communality. Having others around may confer safety or the ability to partially opt out of the experience when challenged. This is consistent with work showing controlled settings are predictors of subjective effects (Carhart‐Harris et al. 2018) and highlighting the importance of support, guidance, and stability (Swanson 2018). One report described how sensory overstimulation and environmental unpredictability could provide affective distress, “the engine revs filled my sensitive shroomed out ears with a demonic despair… I started yelling over the motor for him to stop…he didn't notice” (Report 2). In this instance, the combination of transitioning through different environments and overstimulation could degrade the affective quality of the experience (noting that distress is not the issue so much as being left in prolonged distress alone). Although this did not (in this case) cause lasting harm, it does point to the importance of stability and support in the environment during psychedelic experiences.

### 3.3 After

#### 3.3.1 Theme 1: Experiential Impact (Change in Attitude or Behavior)

A generated theme, experiential impact, reflected how experiences prompted shifts in outlook or behavior and was often eudaimonic. Such changes were linked with subjective aspects of the acute‐phase‐enabled resolution of difficult emotions or behaviors, “I was happy, scared, terrified, and humbled. Most of what I learned on this trip came at the second‐to‐last ‘high’” (Report 19), and “I feel like I no longer live for the future or I am no longer stuck in the past. I feel like I have achieved nirvana” (Report 20).

For example, Reporter 6 seemed to characterize the impact of the experience as eudaimonic, where a rapid and profound positive change occurred in their life:

I hide it well but i was suicidal at the time and have been struggling with depression…it's crazy how it has changed my outlook on the world. I can't say it cured my depression or anything like that. But in my good moments I feel like i'm exactly where i'm supposed to be and I made the decision to be alive. The world is endless and i'm free to do whatever I want. I haven't taken acid since but I don't regret taking it. “Definitely don't consider it a bad trip”. This sense of eudaimonia seemingly altered participants’ assumptions about themselves and their place in the world, arising from meaningful experiential journeys characterized by profound insight. Psychological growth stemmed from relinquishing unhelpful attachments, and re‐evaluation of preexisting psychological concerns. Eudaimonic experiences were a recurrent and even central aspect of subjective judgements of the experience.

## 4 Discussion

Clinical psychedelic outcomes are increasingly well understood, but less is known about naturalistic experiences. Using online forum data, we identified themes within discrete temporal phases that correspond to set and setting components, representing a novel mapping of naturalistic psychedelic experiences into discrete sequential stages. These findings can inform investigations of preparatory, integration, and set/setting mechanisms for controlled psychedelic administration.

Our findings could inform suitability for psychedelic therapy and associated risks, given that tailored knowledge supports willingness and readiness (McAlpine et al. 2024), and that knowledge informs preparation and positive intentions (Danforth 2009; Hartogsohn 2017; Timmermann and Watts 2022). Our theme on experiential aids is consistent with quantitative research (Gandy et al. 2020; Jerotic, Vuust, & Kringelbach, 2023; Kaelen et al. 2018; Kettner et al. 2018).

The emergence of sensory and cognitive distortions aligns with research showing that psychedelics confer insights (McGovern et al. 2024, Gandy et al. 2022; Tulver et al. 2023), leading to belief changes (McGovern et al. 2022; Timmermann et al. 2021). The mindset and affective quality theme, particularly self‐world dissolution, reflects known subjective alterations (Letheby and Gerrans 2017). Environmental stability findings are supported by quantitative research (Golden et al. 2022).

Finally, the theme of experiential impact is consistent with quantitative and theoretical work (Dourron et al. 2022; Letheby and Mattu 2022). Psychedelics may solicit this process of psychological modification, with appropriate experiential or environmental elements being guardrails that guide the person toward experiencing positive (i.e., mental or behavioral) effects. Relevant here is the notion of eustress (i.e., stress which promotes psychological growth) wherein initial difficulties may lead to meaningful and transformative experiences. This was evident in several reports and, as noted above, appeared partly reliant on the use of experiential aids. Our findings are consistent with the idea that appropriate support and contextual stability can shift challenging psychedelic experiences into opportunities for insight and integration. More generally, our findings support the notion that, in contradistinction to traditional psychiatric frameworks that seek to contain or control emotional states, psychedelics may instead allow one to surrender to them. In turn, this may allow people to “be with” emotions, rather than seeking to numb or suppress them, allowing an increased connection to emotions and inner material, and thus promote healing through presence rather than avoidance (Yaden and Griffiths 2020).

### 4.1 Limitations and Future Directions

One limitation is that, given the archival nature of the data, we could not capture how aspects such as neurodivergence, cultural considerations, or systemic barriers may shape or mold psychedelic experience, a shortcoming that future work could focus on more directly. Another important point concerns the notion of critical periods, wherein psychedelics acutely increase capacity for learning (e.g., Nardou et al. 2023). This necessitates the importance of adopting clinical frameworks for integrating insights accrued during the experience (Timmermann and Watts 2022; McGovern et al. 2024), an aspect that is an important focus for future work. Another important aspect for future work is that reports are self‐sourced in preparation and integration. Online communities appeared to function as grassroots harm‐reduction platforms without more formal and expensive services (Pestana, Beccaria, & Petrilli, 2021), reflecting a broader shift toward self‐empowered and peer‐led initiatives. It is important that this shift considers the risks of unregulated practice and the lack of trained facilitators to guide psychedelic journeys. Such unregulated spaces pose risks of culturally appropriative practices (George et al. 2020). This points to the more general importance of accessible harm‐reduction resources (for nonclinical settings) and the need for integrating resources at the community level. To build on our findings, future research could aim to examine how informal support structures might be implemented and strengthened.

## 5 Conclusion

We identified key themes of psychedelic experiences through reflexive thematic analysis. These themes were knowledge, intentionality, and aids before the experience; sensory/cognitive shifts, mindset, and environmental stability during; and psychological change after. These findings note how set and setting can shape a naturalistic psychedelic experience. Understanding the diverse phenomenology of naturalistic psychedelic consumption can inform broader preparation and integration processes. Our findings offer insights into eudaimonic psychedelic experiences from a phenomenological rather than strictly clinical perspective.

## Author Contributions

**H. T. McGovern**: conceptualization, methodology, investigation, supervision, project administration, writing – original draft, writing – review and editing. **L. Bajo**: methodology, software, data curation, formal analysis. **G. Hassed**: methodology, software, data curation, formal analysis. **R. Hoefnagels**: software, methodology, data curation, formal analysis. **M. Levey**: methodology, software, data curation, formal analysis. **N. Rose**: methodology, software, data curation, formal analysis. **A. De Foe**: conceptualization, writing – review and editing. **B. T. Hutchinson**: writing – review and editing, supervision, validation.

## Conflicts of Interest

The authors declare no conflicts of interest.

## Peer Review

The peer review history for this article is available at https://publons.com/publon/10.1002/brb3.70687.

## Supporting information



Supplementary materials

## Data Availability

Data used for the generation of themes in this report are available upon request.

## Endnotes

1 Subreddits included in the study were r/Psychonaut, r/Shrooms, r/LSD, r/Acid, and r/Tripreports.

## References

[brb370687-bib-0001] Aday, J. S. , A. K. Davis , C. M. Mitzkovitz , E. K. Bloesch , and C. C. Davoli . 2021. “Predicting Reactions to Psychedelic Drugs: A Systematic Review of States and Traits Related to Acute Drug Effects.” ACS Pharmacology & Translational Science 4, no. 2: 424–435. 33860172 10.1021/acsptsci.1c00014 PMC8033773

[brb370687-bib-0003] Bienemann, B. , N. S. Ruschel , M. L. Campos , M. A. Negreiros , and D. C. Mograbi . 2020. “Self‐Reported Negative Outcomes of Psilocybin Users: A Quantitative Textual Analysis.” PLoS ONE 15, no. 2: e0229067. 32084160 10.1371/journal.pone.0229067 PMC7034876

[brb370687-bib-0004] Braun, V. , and V. Clarke . 2006. “Using Thematic Analysis in Psychology.” Qualitative Research in Psychology 3, no. 2: 77–101.

[brb370687-bib-0005] Braun, V. , and V. Clarke . 2021. “To Saturate or Not to Saturate? Questioning Data Saturation as a Useful Concept for Thematic Analysis and Sample‐Size Rationales.” Qualitative Research in Sport, Exercise and Health 13, no. 2: 201–216.

[brb370687-bib-0009] Carhart‐Harris, R. L. , L. Roseman , E. Haijen , et al. 2018. “Psychedelics and the Essential Importance of Context.” Journal of Psychopharmacology 32, no. 7: 725–731. 29446697 10.1177/0269881118754710

[brb370687-bib-0010] Denscombe, M. 2017. EBOOK: The Good Research Guide: For Small‐Scale Social Research Projects. McGraw‐Hill Education (UK).

[brb370687-bib-0011] Dourron, H. M. , C. Strauss , and P. S. Hendricks . 2022. “Self‐Entropic Broadening Theory: Toward a New Understanding of Self and Behavior Change Informed by Psychedelics and Psychosis.” Pharmacological Reviews 74, no. 4: 984–1029. 10.1124/pharmrev.121.000514 36113878

[brb370687-bib-0012] Gandy, S. , V. Bonnelle , E. Jacobs , et al. 2022. “Psychedelics as Potential Catalysts of Scientific Creativity and Insight.” Drug Science, Policy and Law 8: 20503245221097649.

[brb370687-bib-0013] Gandy, S. , M. Forstmann , R. L. Carhart‐Harris , et al. 2020. “The Potential Synergistic Effects Between Psychedelic Administration and Nature Contact for the Improvement of Mental Health.” Health Psychology Open 7, no. 2: 2055102920978123. 33335742 10.1177/2055102920978123 PMC7724423

[brb370687-bib-0014] Golden, T. L. , S. Magsamen , C. C. Sandu , et al. 2022. “Effects of Setting on Psychedelic Experiences, Therapies, and Outcomes: A Rapid Scoping Review of the Literature.” Disruptive Psychopharmacology 56: 35–70. 10.1007/7854_2021_298 35138585

[brb370687-bib-0015] Hadar, A. , J. David , N. Shalit , et al. 2023. “The Psychedelic Renaissance in Clinical Research: A Bibliometric Analysis of Three Decades of Human Studies With Psychedelics.” Journal of Psychoactive Drugs 55, no. 1: 1–10. 35000572 10.1080/02791072.2021.2022254

[brb370687-bib-0016] Haijen, E. C. , M. Kaelen , L. Roseman , et al. 2018. “Predicting Responses to Psychedelics: A Prospective Study.” Frontiers in Pharmacology 9: 897. 30450045 10.3389/fphar.2018.00897 PMC6225734

[brb370687-bib-0017] Hartogsohn, I. 2017. “Constructing Drug Effects: A History of Set and Setting.” Drug Science, Policy and Law 3: 2050324516683325.

[brb370687-bib-0019] Kaelen, M. , B. Giribaldi , J. Raine , et al. 2018. “The Hidden Therapist: Evidence for a Central Role of Music in Psychedelic Therapy.” Psychopharmacology 235: 505–519. 29396616 10.1007/s00213-017-4820-5 PMC5893695

[brb370687-bib-0021] Kettner, H. , N. L. Mason , and K. P. Kuypers . 2019. “Motives for Classical and Novel Psychoactive Substances Use in Psychedelic Polydrug Users.” Contemporary Drug Problems 46, no. 3: 304–320.

[brb370687-bib-0024] Kruger, D. J. , O. Enghoff , M. Herberholz , et al. 2023. “"How Do I Learn More about This?": Utilization and Trust of Psychedelic Information Sources among People Naturalistically Using Psychedelics.” Journal of Psychoactive Drugs 55, no. 5: 631–639. 37078418 10.1080/02791072.2023.2201263

[brb370687-bib-0025] Leary, T. , G. H. Litwin , and R. Metzner . 1963. “Reactions to Psilocybjn Administered in a Supportive Environment.” The Journal of Nervous and Mental Disease 137, no. 6: 561–573. 14087676 10.1097/00005053-196312000-00007

[brb370687-bib-0026] Letheby, C. , and P. Gerrans . 2017. “Self Unbound: Ego Dissolution in Psychedelic Experience.” Neuroscience of Consciousness 2017, no. 1: nix016. 30042848 10.1093/nc/nix016 PMC6007152

[brb370687-bib-0027] McAlpine, R. , G. Blackburne , and S. Kamboj . 2024. “Development and Psychometric Validation of a Novel Scale for Measuring ‘Psychedelic Preparedness’.” Scientific Reports 14: 3280. 38332334 10.1038/s41598-024-53829-z PMC10853197

[brb370687-bib-0028] McCartney, A. M. , H. T. McGovern , and A. De Foe . 2023. “Predictors of Psychedelic Experience: A Thematic Analysis.” Journal of Psychoactive Drugs 55, no. 4: 411–419. 36197103 10.1080/02791072.2022.2129885

[brb370687-bib-0029] McGovern, H. T. , H. J. Grimmer , M. K. Doss , et al. 2024. “An Integrated Theory of False Insights and Beliefs Under Psychedelics.” Communications Psychology 2, no. 1: 69. 39242747 10.1038/s44271-024-00120-6 PMC11332244

[brb370687-bib-0030] McGovern, H. T. , P. Leptourgos , B. T. Hutchinson , and P. R. Corlett . 2022. “Do Psychedelics Change Beliefs?.” Psychopharmacology 239, no. 6: 1809–1821. 35507071 10.1007/s00213-022-06153-1

[brb370687-bib-0031] Nayak, S. M. , N. Gukasyan , F. S. Barrett , et al. 2021. “Classic Psychedelic Coadministration With Lithium, but Not Lamotrigine, Is Associated With Seizures: An Analysis of Online Psychedelic Experience Reports.” Pharmacopsychiatry 54, no. 05: 240–245. 34348413 10.1055/a-1524-2794

[brb370687-bib-0032] Nelson, J. 2017. “Using Conceptual Depth Criteria: Addressing the Challenge of Reaching Saturation in Qualitative Research.” Qualitative Research 17, no. 5: 554–570.

[brb370687-bib-0033] Noorani, T. 2021. “Containment Matters: Set and Setting in Contemporary Psychedelic Psychiatry.” Philosophy, Psychiatry, & Psychology 28, no. 3: 201–216.

[brb370687-bib-0035] Pronovost‐Morgan, C. , I. Hartogsohn , and J. G. Ramaekers . 2023. “Harnessing Placebo: Lessons From Psychedelic Science.” Journal of Psychopharmacology 37, no. 9: 866–875. 37392012 10.1177/02698811231182602 PMC10481630

[brb370687-bib-0036] Roberts, C. A. , I. Osborne‐Miller , J. Cole , et al. 2020. “Perceived Harm, Motivations for Use and Subjective Experiences of Recreational Psychedelic 'Magic' Mushroom Use.” Journal of Psychopharmacology 34, no. 9: 999–1007. 32674668 10.1177/0269881120936508

[brb370687-bib-0037] Schlag, A. K. , J. Aday , I. Salam , et al. 2022. “Adverse Effects of Psychedelics: From Anecdotes and Misinformation to Systematic Science.” Journal of Psychopharmacology 36, no. 3: 258–272. 35107059 10.1177/02698811211069100 PMC8905125

[brb370687-bib-0038] Swanson, L. R. 2018. “Unifying Theories of Psychedelic Drug Effects.” Frontiers in Pharmacology 9: 172. 29568270 10.3389/fphar.2018.00172 PMC5853825

[brb370687-bib-0039] Thal, S. B. , L. B. Engel , and S. J. Bright . 2022. “Sober Sitter or Coconsumer? Psychedelics, Online Forums and Preferences for Interpersonal Interactions.” Addiction Research & Theory 30, no. 5: 382–390.

[brb370687-bib-0040] Timmermann, C. , H. Kettner , C. Letheby , et al. 2021. “Psychedelics Alter Metaphysical Beliefs.” Scientific Reports 11, no. 1: 22166. 34815421 10.1038/s41598-021-01209-2 PMC8611059

[brb370687-bib-0041] Timmermann, C. , R. Watts , and D. Dupuis . 2022. “Towards Psychedelic Apprenticeship: Developing a Gentle Touch for the Mediation and Validation of Psychedelic‐Induced Insights and Revelations.” Transcultural Psychiatry 59, no. 5: 691–704. 35313754 10.1177/13634615221082796 PMC9660279

[brb370687-bib-0042] Tulver, K. , K. K. Kaup , R. Laukkonen , et al. 2023. “Restructuring Insight: An Integrative Review of Insight in Problem‐Solving, Meditation, Psychotherapy, Delusions and Psychedelics.” Consciousness and Cognition 110: 103494. 36913839 10.1016/j.concog.2023.103494

[brb370687-bib-0044] Vollenweider, F. X. , and K. H. Preller . 2020. “Psychedelic Drugs: Neurobiology and Potential for Treatment of Psychiatric Disorders.” Nature Reviews Neuroscience 21, no. 11: 611–624. 32929261 10.1038/s41583-020-0367-2

