# Supplementary material for: Deconstructing Psychedelic Phenomenology: A Thematic Analysis of Discrete Phases of the Psychedelic Experience
Source: Brain Behav. 2025 Jul 21;15(7):e70687. doi: 10.1002/brb3.70687 (PMC12277650; doi:10.1002/brb3.70687)
Supplement: Supplementary file 1 — Supplementary materials [file BRB3-15-e70687-s001.docx]

**Supplementary Materials**

**Data Collection Procedure**

Erowid and Effect Index were initially included in our sampling but were later excluded (the former due to requiring permission to reproduce/use reports and the latter due to lack of relevant data) ^[[1]](#footnote-1)^. User agreements indicating these data are publicly available is available in the user agreement on each of the forum websites used in this paper. A convenience sampling approach was used to identify appropriate reports (i.e., the first reports which met criteria for inclusion were included) against our inclusion criteria. Inclusion criteria were adopted favouring in-depth reports for a richer exploration of the data. For inclusion, each post must have 1) described an experience within ten years from the time of experience to the user post, 2) described an experience where the psychedelic was not intended only for medical purposes (i.e., was not prescribed by a doctor), 3) described an experience where no supplementary substance or drug (including another psychedelic) was consumed, 4) written between 500-4000 words, and 5) demarcated into clear categories of “before”, “during”, and “after” experience in order to understand how outcome factors may relate to preparatory and reflection post-experience beyond the acute phase..

If a post was deemed relevant based on its title, the full post was assessed for inclusion, where a post was included for analysis if it met inclusion criteria. A total of 240 posts across databases were included, and we analysed the first 21 that met all criteria, and thus these posts were analysed (i.e., we decided not to scan more reports as the data in our view had reached saturation, see below). Data collection ceased in line with the notion of data saturation - once no new information was being produced from further report analysis (Braun & Clark, 2021). Researchers focusing on each database stopped data collection once repetitive information appeared to arise in subsequent reports.

We note that this notion of saturation has been critiqued (Braun & Clarke, 2021), partially due to the assumption that a pre-determined fixed point would be sufficient for deep understanding. We thus need to acknowledge that further exploration of our reports could yield an even deeper understanding. By saturation, then, we mean that the data reached a sufficient degree of conceptual depth (Nelson, 2017) or richness and meaningfulness being achieved (see Braun & Clarke, 2022).

It is not uncommon for qualitative analyses to range in the scope of 15-25 reports, which is concordant with our selection of the data. While it could be critiqued that a larger selection would yield more robust data, the intention of our methodology was to capture a cross-sectional understanding of prevalent themes across what were deemed “high quality” and relatively reliable reports; suggesting that our selection could provide a well-considered indicator of themes arising at broader scale. The fact that we found that after 20 reports, no new information was being noticed, and the fact that this falls within the typical range of reports of this nature, meant we stopped looking at further reports after the first 20 (see Supplementary materials for further justification).

**Assumptions**

We adopted a realist-based-experiential approach. Experiential approaches consider language as reflective of the true nature of (in this case) participants truth or experienced reality (Braun & Clarke, 2013). As we aimed to stay faithful to the way in which participants made sense of the experience, we veered toward a semantic approach in how data was explored. In other words, (and consistent with a realist framework), we assumed that meaning was stated explicitly rather than assuming a deeper subtext, typical of latent approaches (Wiltshire & Ronkainen, 2021). Finally, we combined elements of induction (in the early phases) and deduction (i.e., theory informed our approach at later phases) in the generation of themes.

**Data**

Online forums lend themselves to qualitative analysis for several reasons. Firstly, the richness of online user reports is less restricted than quantitative surveys. Secondly, research suggests anonymity facilitates increased disclosure (Clark-Gordon et al., 2019; Hollenbaugh & Everett, 2013), meaning a potentially more ‘honest’ dataset. Finally, the length of the reports and the strict inclusion criteria (see below) allow for breadth and depth of the data.

The publicly accessible online databases we sampled from act as community-supported forums that offer access to user-generated posts of psychoactive substance use experiences. These forums serve as useful hubs for the collection of data for psychedelic consumption in non-clinical settings (Nayak et al., 2021; Thal, Engel, & Bright, 2022). In each post, the person describes aspects of preparation, acute psychedelic experience, and their evaluations of the experiential quality and psychological effects of their psychedelic ingestion. These online forums offer advantages for online qualitative research given they contain a high degree of traffic by active users, and are moderated for content (Denscombe, 2017). Notably, the diversity of forums we sampled from offers an advantage in that it protects against the limitations/biases of any single forum and therefore provides a more heterogenous sample (Fassinger, 2005) of perspectives and insights in the community. We thus used purposive sampling, in that our data were purposely selected to be as information rich as possible (Patton, 2014).

**Analysis Procedure**

Posts were analysed using NVivo v. 12 (Castleberry, 2014). A reflexive thematic analysis was conducted using an adaptation of Braun and Clarke’s (2006) protocol (see *Figure 1*) distributed across five researchers. This method was appropriate given its theoretical flexibility: the analysis was partly confirmatory, and partially exploratory as themes inside these groupings were not predetermined, allowing them to be constructed from the retrieved data set. Thematic analysis of reports was conducted in six phases as described in Braun and Clarke’s (2006) analytic protocol (see *Figure 1*). To solicit a deep understanding of themes, we iterated the final three phases several times.

***Figure 1.*** *Depiction of the adapted Braun and Clarke thematic analysis utilised for developing and labelling themes. This figure denotes an example of our funnelling process from phases 1 through 5, with the legend on the bottom right of the figure illustrating which bits of information were merged, retained, discarded, or reiterated (see below for details). Note that in this context, “merged” refers to combining similar/relevant themes into a reflective unitary theme, whilst “reiteration” refers to refinement of relevant theme or code.*


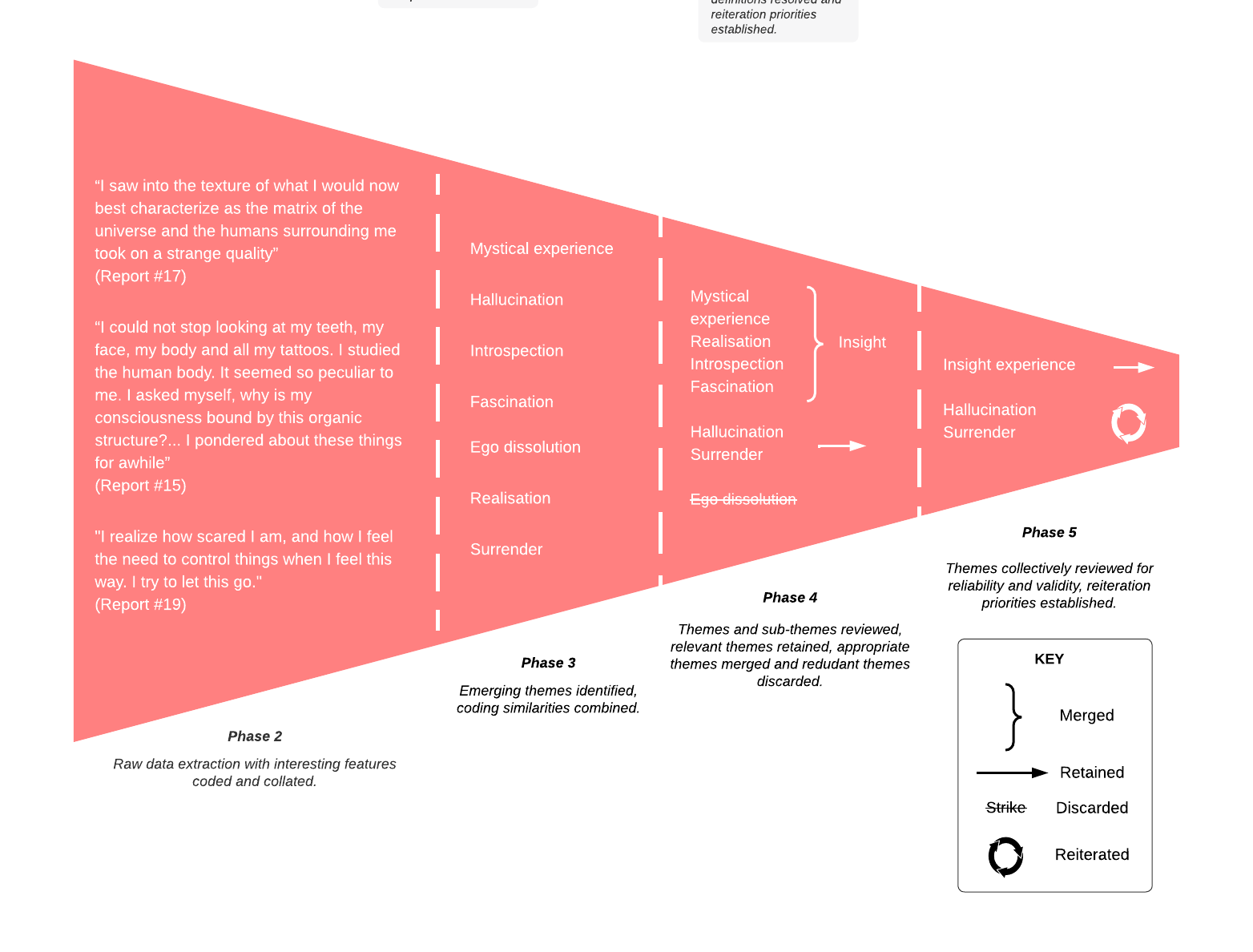


B)

In Phase 1, familiarisation with the data and its narratives was conducted, with each researcher analysing their accounts independently. Within this phase, a reliable and consistent format of data structure across reports was identified and labelled. This structure included events occurring “before” (describing information provided prior to onset), “during” (describing events occurring throughout the acute phase), and “after” (describing information provided following the psychedelic experience). Although accounts were presented as one block (without unique database fields to appraise the three stages), researchers were clearly able to demarcate components of the report via linguistic markers (i.e., past, present, future tens language change in the writing).

In Phase 2, preliminary concepts were identified and used to produce preliminary codes through induction and deduction, involving application of previous assumptions (i.e., existing literature, such as that on intentionality, as well asset and setting) to specific report codes (deduction) and overarching conclusions constructed from individual coding instances (induction) (Braun & Clarke, 2006).. Codes were categorised into their respective temporal locations based on the data format previously identified (i.e., before, during, after). Concepts generated from the data set were discussed as a group, with identification of codes with similar or differential thematic backgrounds noted for future exploration (e.g., visual alterations vs. mystical experience).

In Phase 3, theme searching was conducted via a formal grouping of initial codes, done in accordance with their thematic similarity (XXX). Higher-order themes were identified and discussed, with residual themes grouped beneath them. The identified themes were then grouped into temporally relevant locations (i.e., before, during, after) with each report reviewed collectively as a research group. We reiterated between Phases 3, 4, 5, and 6 several times.

In Phase 4, themes were examined by five researchers, bespoke concepts were expanded on, and unnecessary themes were integrated or discarded, allowing identification of distinct and comparable concepts across reports. Sub-themes generated from the dataset as variances in experience were identified and classified under relevant higher-order themes. For consistency across coding, initial conflicts arising surrounding each theme's representation or classification were deliberated amongst the research team until deeper understanding of the theme was collectively established. Disagreements were handled via discussion and eventual voting if disagreement persisted (i.e., three of five researchers voted for the naming or placing of a particular theme). In this phase, a thematic map was generated and redundancies in the coding were also removed (i.e., identified themes that were inconsistent or irrelevant).

In Phase 5, representations of each theme and sub-theme were finalised based on deliberation. This was done reiteratively, whereby newly presenting themes added to the coding framework (Phase 3) and modifications made to the thematic structure (Phase 4) were reviewed, discussed, and confirmed. Where multiple instances of opposing codes were present *within* a report, overall thematic classification was resolved by collectively determining which was more pertinent. Inconsistencies *between* individual reports were examined by five of the seven authors, with mutual agreement established prior to reiterative attempts and were finalised after four occasions. The final two authors then reviewed the presented codes to resolve any outstanding questions or inconsistencies. After this process, and in line with the recursive nature of thematic analyses, the researchers again collectively discussed any overlaps that might exist between themes – so to ensure resultant themes did not risk becoming topic summaries. As such, the researchers recursively moved between phases 4, 5, and 6 to ensure a deep exploration of themes. Finally, two researchers discussed appropriate naming and use of generated themes, again making efforts to ensure appropriate depth of thought in theme generation. In Phase 6, final interpretations of the data were reported.

**Figure 1.** Schematic of the process followed and iterated to arrive at themes via reflexive thematic analysis.


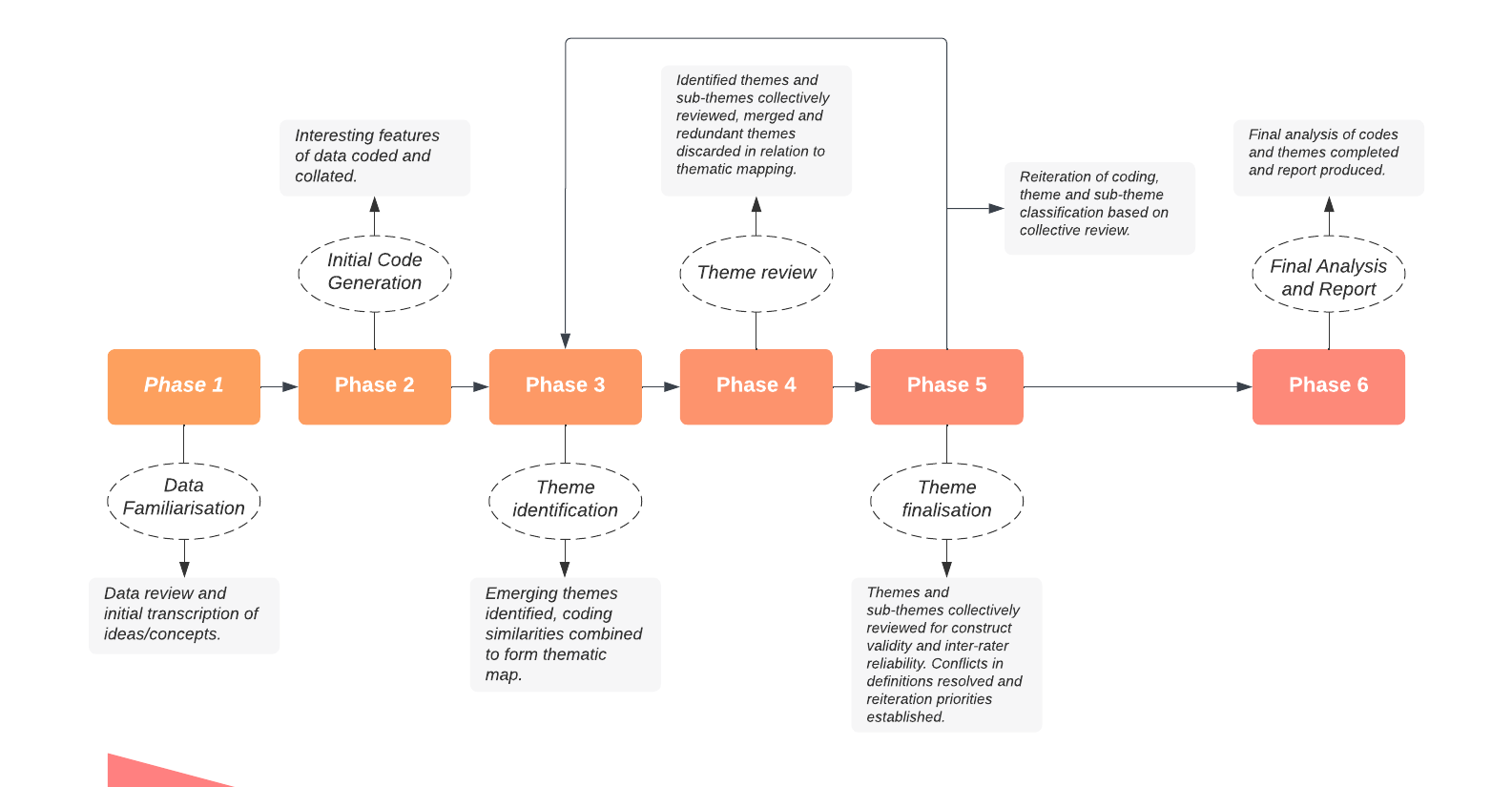


**Figure 2**

*Final framework of generated themes and sub-themes based on temporal location, where there are three temporal phases – before, during, and after the acute phase. The first phase ‘Before’ refers to themes before psychedelic ingestion, parsed into set and setting. “During” refers to themes generated during the acute phase (both internal and external factors). The “After” epoch refers to the themes generated after the experience, about the experience.*


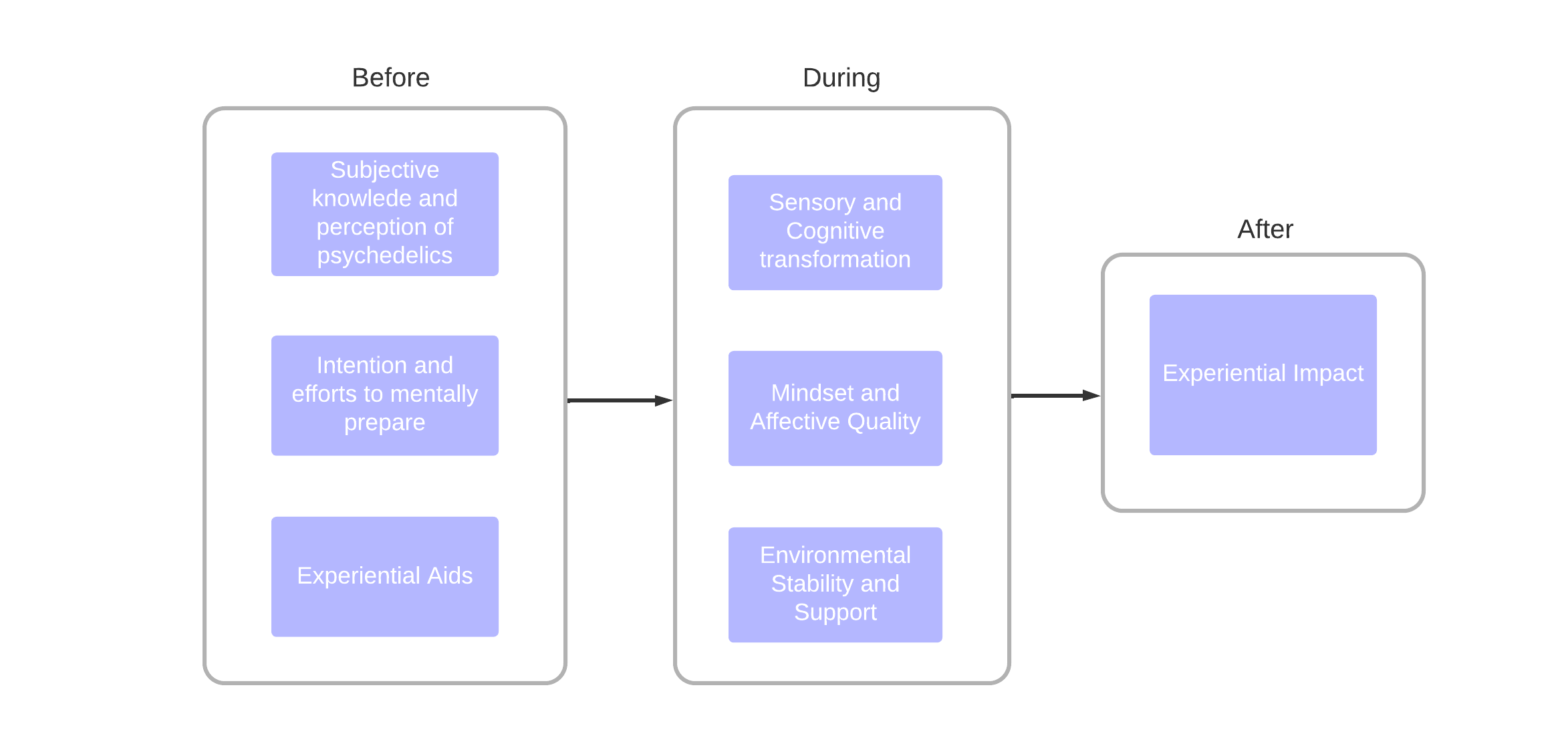


1. Erowid were contacted to ask for the relevant permissions, but no response was received. [↑](#footnote-ref-1)
